# Building the Foundation for Standardized Care Metrics in Jejunoileal Atresia: A Systematic Review of Reported Baseline Characteristics, Treatment Variables and Outcomes

**DOI:** 10.3390/jcm14165693

**Published:** 2025-08-12

**Authors:** Linde Margriet van der Kamp, Cristina Moglia, Enrico La Pergola, Daniel Rossi, Nadine Maria Teunissen, Lucia Migliazza, René Maria Henricus Wijnen

**Affiliations:** 1 European Reference Network for Rare Inherited and Congenital Anomalies (ERNICA), Dr. Molewaterplein 40, 3015 GD Rotterdam, The Netherlands; 2 Department of Pediatric Surgery, Erasmus MC Sophia Children’s Hospital, Erasmus University Medical Center Rotterdam, Dr. Molewaterplein 40, 3015 GD Rotterdam, The Netherlands; 3 Dutch Institute for Clinical Auditing (DICA), Rijnsburgerweg 10, 2333 AA Leiden, The Netherlands; 4 Department of Pediatric Surgery, Azienda Ospedaliera Papa Giovanni XXIII, Piazza OMS 1, 24127 Bergamo, Italy; 5 Department of Pediatric Surgery, Azienda Ospedaliera di Padova, Via Nicolò Giustiniani 2, 35128 Padova, Italy; 6 Department of Women’s and Children’s Health, Karolinska Institutet, Tomtebodavägen 18A, 171 77 Stockholm, Sweden

**Keywords:** Jejunoileal atresia, intestinal, outcome, patient characteristics, quality indicator set

## Abstract

**Background/Objectives**: An evidence-based list of key variables regarding jejunoileal atresia (JIA) care needs to be established to enable quality evaluation and optimization of its care. The aim of this study is to provide an overview of reported patient, treatment, and outcome variables for JIA as documented in recent literature. This list has not been developed previously and will be the foundation for a JIA quality indicator set of the European Pediatric Surgery Audit (EPSA). **Methods**: A systematic review of the literature on the primary care path of JIA, published between 2013 and 2023, was conducted following the PRISMA guidelines. All relevant patient characteristics, parameters regarding JIA treatment, and outcomes were extracted from the included publications. **Results**: A total of 844 variables were extracted from 94 included articles. One hundred fifty-seven parameters were mentioned in more than 5% of publications. The most mentioned patient characteristics were sex (86%), gestational age (71%), and associated anomalies (66%). The most mentioned treatment parameters were stoma placement (34%), primary anastomosis (41%), and time to full enteral nutrition (24%). Most mentioned outcomes were mortality (70%), length of hospital stay (55%), and complications (60%). **Conclusions**: This study created an overview of reported patient characteristics, treatment, and outcome variables regarding the treatment of JIA. A focus on the short-term management and outcomes of JIA was observed; frequently discussed topics were perioperative management, surgical techniques, and feeding management. Our results will serve as the foundation for a Delphi study to develop a core indicator set for JIA, enabling benchmarking and measurement of quality of care.

## 1. Introduction

Intestinal obstruction is one of the main causes of surgical emergencies during the neonatal period [1]. It can be caused by various, often congenital conditions. When obstruction is not promptly recognized, neonates can deteriorate rapidly, resulting in increased morbidity and mortality. Additionally, the required surgical intervention may be more challenging after delayed diagnosis and carry a higher risk of adverse outcomes [2].

**Figure 1:**
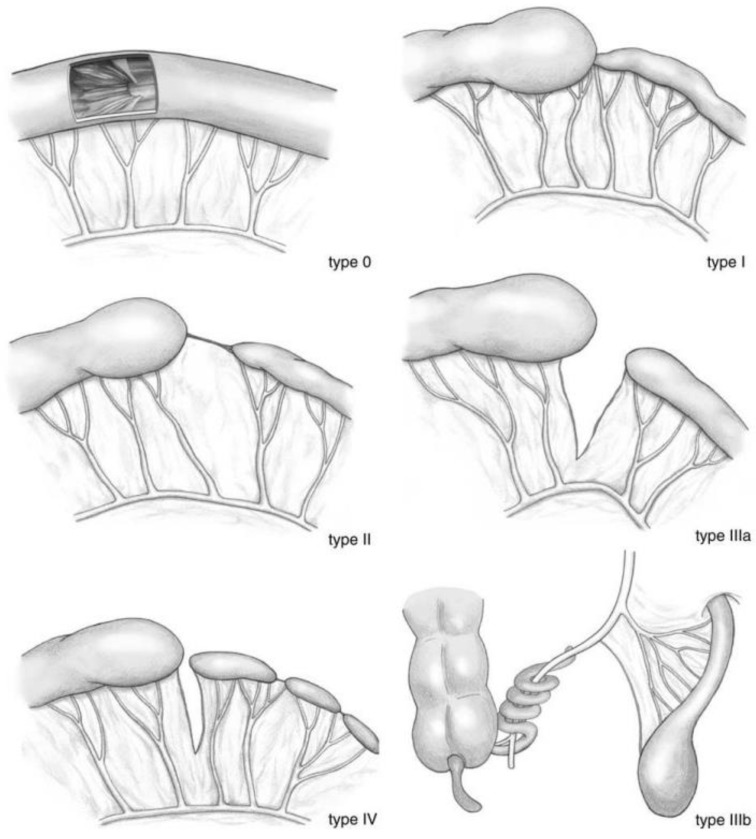
Modified Martin and Zerella classification of jejunoileal atresia by Stolman [8].

One of the major causes of congenital intestinal obstruction is jejunoileal atresia (JIA) [3]. This congenital anomaly occurs in 0.87 per 10,000 births, including live births and still births [4]. Various theories exist regarding the etiology of JIA. One theory suggests that a prenatal vascular event disrupts blood supply to the developing intestine, causing ischemic damage, ultimately leading to intestinal atresia [5]. A second theory proposes that the fusion of vacuoles with the developing intestinal mucosal epithelium is critical in the formation of the gut during embryonic development. Disruptions in this process can result in intestinal atresia [6]. A third theory poses that prenatal volvulus may lead to intestinal loss and the development of atresia [7]. The severity of JIA is categorized using various similar categorization systems, such as Louw’s classification and Grosfeld’s modification or the Martin and Zerella classification; Figure 1 shows an example of the slightly modified classification of Martin and Zerella by Stolman (Figure 1) [8]. Type IIIb, also known as apple peel atresia, involves atresia beyond the duodenojejunal flexure, coiling of the remaining small bowel around the ileocolic artery, and absence of the superior mesenteric artery beyond the origin of the middle colic vessel. Types III and IV are associated with significant loss of bowel length, which increases the likelihood of comorbidities [7].

Despite the seriousness of this condition, mortality steadily declined during the 20th century. Contributing factors include improved pre- and postnatal diagnostics, use of total parenteral nutrition, improved anesthetic and surgical techniques, as well as improvements in the neonatal intensive care unit [5]. In recent years, mortality rates stabilized at approximately 10% [2,8]. While mortality has declined, the incidence of late complications has increased [8]. This rise could be attributed to higher survival rates, resulting in patients with comorbidities during childhood and later in life. Such a transition from mortality to morbidity not only allows for but also demands a shift in focus; alongside efforts to decrease mortality, it is essential to better understand morbidity and longer-term outcomes. Additionally, evaluating the quality of care is crucial to improving both the care process and these long-term outcomes.

A tool that can be used to evaluate and improve the quality of care is clinical auditing, which is defined as “the systematic analysis of processes and outcomes of medical care with the ultimate aim of improvement” [9]. The European Pediatric Surgical Audit (EPSA) is such a clinical audit. Quality indicator sets are used to enable measurement and benchmarking of the quality of care. Various quality indicator sets have been developed for conditions with a higher prevalence [10,11]. However, because clinical auditing is uncommon in rare diseases due to low patient numbers and variation in treatment, such quality indicator sets often do not exist [12]. Recently, a small number of core indicator sets for rare diseases have been developed, including Esophageal atresia and Hirschsprung’s disease [12,13]. These sets were implemented in the EPSA and are used for European benchmarking and measurement of quality of care. To our knowledge, no quality indicators for JIA care have ever been developed.

To develop quality indicators, it is crucial to identify key variables in the care process that reflect differences in care and can be used to assess its quality. In this study, we aim to create an overview of reported outcome variables as well as variables regarding the treatment process of JIA, as reported in recent literature. Furthermore, we will collect patient characteristics to gain insight into the patient population and to enable the development of case-mix models. The resulting list of collected items will subsequently be used as a base for a Delphi study to eventually create a core indicator set for intestinal atresia. This core set of quality indicators will be implemented in the EPSA, allowing for measurement and benchmarking of care quality, ultimately assisting in identifying areas for improvement and enhancing patient outcomes.

## 2. Materials and Methods

This review was performed according to the Preferred Reporting Items for Systematic Reviews and Meta-analysis (PRISMA) statement and guidelines [14]. A search strategy for Medline, Embase, and the Cochrane Library was developed in collaboration with a medical librarian from the Erasmus Medical Center. This search was based on the search term “intestinal” or “jejunoileal atresia” combined with the terms “treatment outcome”, “mortality”, “morbidity”, “complication”, “reoperation”, and “short bowel syndrome”. Complete search strategies are provided in Supplementary Materials S1—Search strategy. The search was performed in July 2023.

### 2.1. Inclusion and Exclusion Criteria

All publications related to the main care process of jejunoileal atresia patients were included, thus also including articles regarding longer-term follow-up. Papers published before 2013 were excluded to ensure contemporaneity. Publications focusing on colonic, duodenal, or biliary atresia were excluded, as well as those addressing the care path of other rare congenital anomalies in which intestinal atresia was described solely as an associated anomaly. Additional exclusion criteria included the following: non-English-language publications, in vitro or animal studies, case series with fewer than five patients, editorials, letters, meeting abstracts, reviews, and epidemiological studies.

### 2.2. Selection Process

L.M.K., C.M., and E.L.P. independently reviewed the titles and abstracts of all search results to assess their relevance to the reporting of management and outcomes in patients with jejunoileal atresia. Subsequently, the full texts of included articles were screened to confirm this relevance. Disagreements were discussed, and if necessary, resolved by R.W. and L.M. The reviewing authors were not blinded to the journal name, title, or authors.

### 2.3. Data Extraction, Analysis, and Results

Data extraction was facilitated using an Excel-based framework. The origin, year of publication, study design, and the number of included patients in each study were documented. Parameters describing the care path, outcomes, and patient characteristics were extracted from the included articles and subsequently collected and categorized in a separate file. Similar variables were merged following consensus among reviewers. Parameters mentioned in more than 5% of included publications were added to the framework and reviewed by two independent reviewers. This threshold was chosen to avoid excluding potentially relevant variables due to inconsistent reporting practices, which are common in the context of limited and heterogeneous data in rare diseases such as JIA. This approach aligns with thresholds applied in previous systematic reviews on similar conditions, such as esophageal atresia and Hirschsprung’s disease [12,13]. This review did not entail the assessment of methodological quality of the included papers, nor the extraction or interpretation of parameter estimates.

## 3. Results

### 3.1. Included Publications

**Figure 2:**
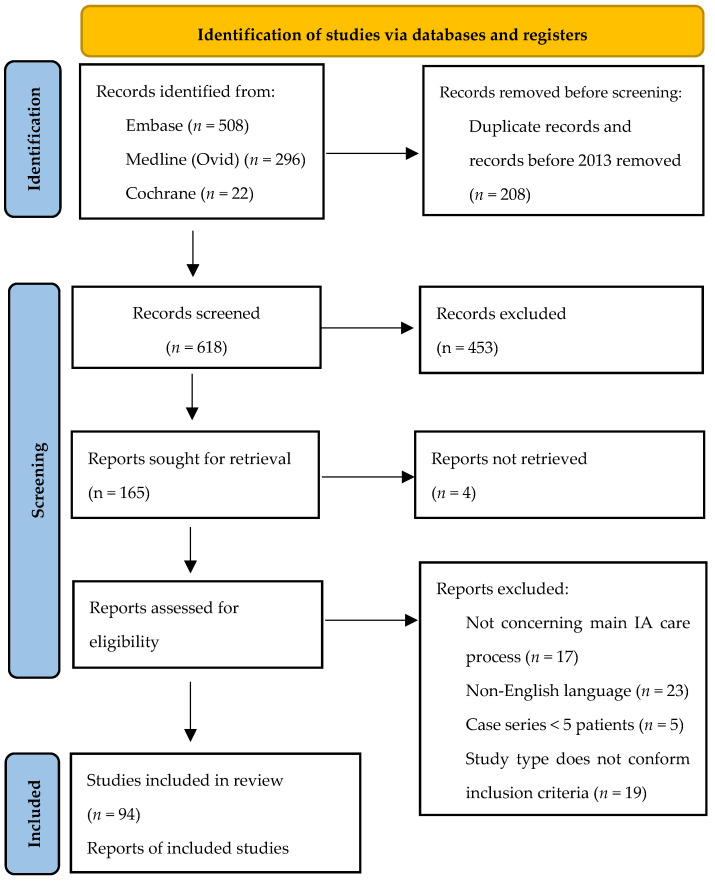
PRISMA flow chart. Systematic review of studied parameters in jejunoileal atresia.

The search strategy identified 826 publications. After the removal of duplicates and limiting the search to articles published between 2013 and July 2023, 618 articles remained, of which 94 met the inclusion criteria (Figure 2). Table 1 summarizes study characteristics, including the number of patients, study location, and period, and study design for all included publications. Most studies were conducted retrospectively (88%), and 65% of studies included fewer than 50 patients. An individualized overview of study characteristics corresponding to the included articles is provided in Table S1.

### 3.2. Data Extraction

A full-text analysis of the 94 articles identified 842 unique parameters described in at least one publication. These parameters were categorized into patient characteristics (*n* = 243), treatment and care process characteristics (*n* = 274), and outcomes (*n* = 325). Then they were further arranged by topic, such as treatment type, complications, or long-term outcomes. The complete overview of included patient characteristics, treatment and care process characteristics, and outcomes is included in Tables S2–S4.

### 3.3. Extracted Parameters

Of the 842 identified parameters, 157 were mentioned in more than 5% of included publications. Table 2 shows an overview of patient characteristics, Table 3 shows structure and process variables, and Table 4 shows outcomes mentioned in more than 5% of included publications. Patient characteristics that were mentioned most often were sex (86%), gestational age (71%), any associated anomalies (66%), and birth weight (64%). Age at surgery was mentioned in 44% of articles, and the type of atresia following the Gross classification was mentioned in 41% of articles. Treatment and care process characteristics were often related to the type of surgery and feeding, including variables such as placement of a stoma (34%), primary anastomosis (41%), time to full enteral nutrition (24%), and the duration of parenteral nutrition (29%). Duration of follow-up was mentioned in nearly a quarter of the included articles (23%).

Frequently mentioned outcomes were mortality (70%), length of hospital stay (55%), complications (60%), and unplanned reoperation (43%). Because various complications, such as sepsis (51%), anastomotic leakage (38%), and wound infection (30%), were reported separately, these variables were also collected separately, providing insight into the specific complications that were mentioned most frequently, as reported in recent literature.

## 4. Discussion

We aimed to establish an overview of reported patient characteristics, treatment, and outcome variables in intestinal atresia care, as reported in recent literature. A total of 842 parameters were identified from 94 publications, demonstrating a wide variability in research interest, reporting, and definitions. Four patient characteristics—sex, gestational age, associated anomalies, and birth weight—and four outcomes—mortality, length of hospital stay, complications, and the specific complication sepsis—were reported in over 50% of the articles. No variables related to the structure or process of JIA care were mentioned in more than 50% of the articles.

Research on rare diseases is limited because of the low incidence, which makes it challenging to collect data from large patient cohorts, which is necessary when aiming to achieve high statistical power. Most of the studies included (86%) involved patient cohorts of fewer than 100 patients, with 65% including fewer than 50 patients. Additionally, this review demonstrates considerable variability in reported parameters, their timing of measurement, and the definitions used. An example of this is the interchangeable use of the terms “anastomotic stricture” and “anastomotic stenosis”, which, although often used synonymously, technically refer to distinct entities; stricture typically denotes a fibrotic, often irreversible narrowing, while stenosis includes a broader range of potentially reversible causes such as edema or inflammation [15]. However, these can also be difficult to distinguish clinically. This variability severely limits comparability and prevents meta-analyses, data comparison, and benchmarking. To improve the utility of data, standardized data collection and consistent timing of measurements are essential. This will enable larger pooled samples, facilitate meta-analyses, and ultimately enhance the quality of evidence.

This review on jejunoileal atresia demonstrates that most studies were conducted retrospectively (*n* = 83, 88%), with only a limited number of prospective studies included (*n* = 9, 10%). Just one (1%) randomized controlled trial was identified. This finding is unsurprising, given the challenges associated with conducting clinical trials in the pediatric surgical field of congenital malformations, which require individualized treatment strategies. Considering these obstacles, prospective data collection in registries and audits could offer considerable added value, a potential that is increasingly being recognized on a global scale [16]. Moreover, clinical auditing allows for the collection of real-world data, which by nature is representative of the entire patient population, instead of a selected sample size.

The extracted variables primarily focus on short-term aspects of JIA care, including diagnostics, perioperative management, feeding strategies, surgical techniques, and postoperative complications. Fewer studies reported longer-term outcomes, and many developmental outcomes were only recorded once. This imbalance may be attributed to the relative ease of reporting short-term outcomes compared to long-term outcomes. Besides, the shift from mortality to morbidity and long-term outcomes as seen in clinical practice is only gradually taking place in a research setting. The limited evidence on long-term follow-up shows mixed results, particularly regarding the impact of neonatal surgery on neurodevelopmental outcomes [17,18,19,20]. This emphasizes the need for longer-term follow-up research.

Another possible reason for the emphasis on short-term outcomes is the origin of the included studies, many of which come from low- and middle-income countries where JIA care still demands prioritization of short-term outcomes. The study origins also explain the inclusion of variables related to the availability of specific facilities, such as neonatal intensive care units, total parenteral nutrition, or licensed pediatric surgeons. While it is debatable whether these parameters are relevant to European health care, this review includes all the parameters mentioned in JIA literature over the last decade.

Our study has some limitations. To ensure inclusion of parameters representative of current practice, the search was limited from 2013 to 2023. It is possible that extension of the period could have led to the inclusion of more outcomes than in the current research, but we believe that the pool of included articles is representative of the current JIA care practice. Furthermore, 22/165 articles (13%) were excluded due to non-English publications, which may have led to an underestimation of reported variables and practice variation. Lastly, although this study serves as the foundation for a European core quality indicator set, only 18% of the included studies originated from European countries. However, the limited number of European publications and the adaptation of the set through a Delphi study involving European experts justify the inclusion of non-European studies.

## 5. Conclusions

This study developed an overview of reported patient characteristics, treatment, and outcome variables regarding the treatment of JIA, as reported in the recent literature of the last decade. It will serve as the foundation of a Delphi study to develop a core quality indicator set for JIA, which will be implemented in the EPSA. This will enable benchmarking and measurement of the quality of care to support the improvement of JIA care. Future research should prioritize the development and implementation of standardized sets, such as core outcome sets and core quality indicator sets, to enable meaningful comparison across studies and improve the consistency of reporting. In addition, a greater emphasis on long-term follow-up data is essential to gain deeper insights into patients’ nutritional status, physical and cognitive development, bowel function, and overall quality of life. Finally, fostering international collaboration, supporting multicenter studies, and promoting the collection of real-world data through clinical audits and registries will be critical steps toward advancing our understanding and management of JIA.

## Figures and Tables

**Table 1:** Study characteristics of included publications.

		*N*	%
**Originated in**	Africa	16	17
	Asia	40	43
	Central America	1	1
	Europe	17	18
	North America	17	18
	Oceania	1	1
	South America	2	2
**Study timing**	Retrospective	83	88
	Prospective	9	10
	Cross-sectional	2	2
**Type of study**	Observational	62	66
	Comparative	32	34
**Study design**	Cohort	88	94
	Case-control	5	5
	Randomized controlled Trial	1	1
**Year of publication**	2013	2	2
	2014	6	6
	2015	3	3
	2016	12	13
	2017	10	11
	2018	10	11
	2019	16	17
	2020	5	5
	2021	7	7
	2022	15	16
	2023	8	9
**Number of included patients**	<10	11	12
	<25	19	20
	25–50	31	33
	50–100	20	21
	100–300	8	9
	300–1000	2	2
	>1000	3	3

*N* = number of included studies.

**Table 2:** Patient characteristics mentioned in >5% of articles.

Patient Characteristics	*n*	%
Sex	81	86
Gestational age	67	71
Any associated anomalies	62	66
Birth weight (grams)	60	64
Age at surgery (days)	41	44
Type of atresia (I: mucosal web, II atretic fibrous cord, IIIa: V-shaped mesenteric defect, type IIIb apple peel atresia, type IV: multiple atresias)	39	41
Cardiac anomalies	38	40
Prematurity	33	35
Level of obstruction (duodenal, jejuno-ileal, colonic)	31	33
Gastrointestinal anomalies	28	30
Urogenital anomalies	22	23
Site of intestinal atresia (anatomical location of atresia)	21	22
Age at presentation (days)	18	19
Respiratory anomalies	16	17
Anomalies of the Central Nervous System	15	16
Vomiting (presenting symptom)	15	16
Weight at surgery (grams)	14	15
Musculoskeletal anomalies	14	15
Weight at presentation (grams)	13	14
Type of delivery (vaginal or caesarean)	13	14
Abdominal distension (presenting symptom)	13	14
Trisomy 21	13	14
Malrotation of intestine	13	14
Anorectal malformation	12	13
Age at admission (days)	10	11
Maternal age	10	11
Apgar 5 min	9	10
Syndrome	9	10
Volvulus (presenting symptom)	9	10
Venous malformation	9	10
Renal anomalies	9	10
Oesophageal atresia/Tracheoesophageal fistula	9	10
Failure to pass meconium (presenting symptom)	8	9
Chromosomal abnormalities	8	9
Cystic fibrosis	8	9
Annular pancreas	8	9
Race (Caucasian, African-American, Asian, other)	7	7
Apgar 1 min	7	7
ASA class	7	7
Ventricular septal defect	7	7
Gastroschisis	7	7
Location of delivery	6	6
Patent ductus arteriosus	6	6
Meckel diverticulum	6	6
Small for gestational age	5	5
Age at diagnosis (days)	5	5
Primipara vs. multipara	5	5
Duodenal atresia	5	5
VACTERL association	5	5

Abbreviations: VACTERL, vertebral defects, anal atresia, cardiac defects, tracheoesophageal fistula, renal anomalies, and limb abnormalities.

**Table 3:** Structure and process variables mentioned in >5% of articles.

Structure and Process Variables	*n*	%
**Structure**		
Hospital level	25	27
Treatment at a tertiary referral center	20	21
Presence of a neonatal intensive care unit	11	12
Operation performed by (licensed specialized) pediatric surgeon	10	11
Facilities for pediatric ventilation	8	9
Hospital transfer (patient transfer status)	6	6
Location of delivery	6	6
Facilities for total parenteral nutrition	5	5
**Process variables**	***n***	**%**
Performance of a resection	41	44
Primary anastomosis	39	41
Placement of a stoma	32	34
Prenatal diagnosis	27	29
Total duration of parenteral nutrition (in days)	27	29
Duration of procedure	24	26
Time to full enteral nutrition (in days, since operation)	23	24
Duration of follow up	22	23
Type of procedure (laparotomy or laparoscopy)	21	22
Performance of an abdominal X-ray for diagnosis	19	20
Description of the type of resection	19	20
Performance of tapering enteroplasty	18	19
Time to start enteral feeding (in days, since operation)	15	16
Time to initial oral feeding (in days, since operation)	15	16
Description of the surgical technique	15	16
Type of anastomosis (end/end, end/side, side/side)	15	16
Prenatal ultrasound	14	15
Prenatal diagnosis of polyhydramnios	13	14
Type of sutures	12	13
Discrepancy proximal and distal diameter	11	12
Time to establishment of full oral intake	10	11
Time to bowel continuity	10	11
Performance of intravenous fluid replacement at and during admission	10	11
Nasogastric tube placement	10	11
Technique of the anastomosis (e.g., hand sewn, stapler)	9	10
Suturing technique used for the anastomosis	9	10
Intestinal perforation found during operation (intraoperative finding)	9	10
Provision of preoperative antibiotics	9	10
Type of incision	8	9
Malnutrition at admission or during hospital stay	8	9
Performance of laboratory investigations	8	9
Placement of a central line/peripherally inserted central catheter	8	9
Performance of the bishop-Koop procedure	8	9
Performance of an abdominal ultrasound for diagnosis	8	9
Performance of 1-year follow-up	8	9
Duration of mechanical ventilation	7	7
Breast milk use	7	7
Timing of start of parenteral nutrition (age of start, pre- or postoperatively)	6	6
Measurement of serum electrolytes	6	6
Measurement of direct bilirubin	6	6
Location of anastomosis	6	6
Procedure priority (emergency, elective, semi-elective)	6	6
Resuscitation	6	6
Length of resected intestine (cm)	6	6
Age at diagnosis (days)	5	5
Feeding intolerance (postoperative)	5	5
Tripple bubble sign diagnosed (on X-ray)	5	5
Measurement of albumin	5	5
Double bubble sign diagnosed	5	5
Estimated blood loss	5	5
Time between presentation and surgery	5	5
Dilated bowel loops visible (on X-ray)	5	5
Number of anastomoses	5	5

**Table 4:** Outcomes mentioned more than 5 percent.

Outcomes	*n*	%
Survival rate/mortality	66	70
Complications	56	60
Length of hospital stay	52	55
Sepsis	48	51
Unplanned reoperation	40	43
Anastomotic leakage	36	38
Wound infection	28	30
Bowel obstruction	26	28
Anastomotic stricture/stenosis	19	20
Short bowel syndrome	17	18
Remaining small bowel length	17	18
Respiratory infection	17	18
Necrotizing enterocolitis (NEC)	16	17
In-hospital mortality (death before discharge)	13	14
Wound dehiscence	12	13
Episodes of meconium peritonitis	11	12
Central line-associated bloodstream infection (CLABSI)	10	11
Hepatic cholestasis	10	11
Infection (general)	9	10
Liver failure	9	10
Stoma related complications	9	10
Number of operations	8	9
Pneumonia	7	7
Intestinal necrosis	7	7
Readmission	7	7
Short term complications (<30 days)	7	7
Ileus	7	7
Growth	7	7
Postoperative intestinal perforation	6	6
30-day mortality	6	6
Development	6	6
Growth in weight	6	6
Growth in height	6	6
Preservation of ileocecal valve	6	6
Re-anastomosis	6	6
Meconium ileus	6	6
Aspiration pneumonitis	6	6
Stomal prolapse	6	6
Gastrostomy or gastrojejunostomy dependent	5	5
Incisional hernia	5	5
High output stoma	5	5
Development of parenteral nutrition associated liver dysfunction (PNALD)	5	5
Surgical re-exploration and enterostomy	5	5
Urinary tract infection	5	5
Respiratory failure	5	5
Ischemia (intraoperative, primary surgery)	5	5
Postoperative length of hospital stay	5	5

## Data Availability

The original contributions presented in this study are included in the article/Supplementary Materials. Further inquiries can be directed to the corresponding author.

## Supplementary Materials

The following supporting information can be downloaded at https://www.mdpi.com/article/10.3390/jcm14165693/s1: Supplementary materials S1—Search strategy, Table S1: Individualized overview of included studies and study characteristics, Table S2: All identified patient characteristics, Table S3: All identified structure and process characteristics, Table S4: All identified outcomes.

## Abbreviations

The following abbreviations are used in this manuscript:

JIA	Jejunoileal atresia
EPSA	European Pediatric Surgical Audit
