# Supplementary material for: Building the Foundation for Standardized Care Metrics in Jejunoileal Atresia: A Systematic Review of Reported Baseline Characteristics, Treatment Variables and Outcomes
Source: J Clin Med. 2025 Aug 12;14(16):5693. doi: 10.3390/jcm14165693 (PMC12386392; doi:10.3390/jcm14165693)
Supplement: Supplementary file 1 [file jcm-14-05693-s001.zip › jcm-3752219 - Supplementary Table 3 - All identified structure and process characteristics.pdf]

Table S3. All identified structure and process characteristics.

Extracted variables	n	%
**Structure (19)**		
Location of delivery	6	6
Hospital level	25	27
Hospital transfer (patient transfer status)	6	6
Tertiary referral center	20	21
Neonatal intensive care unit	11	12
Nurse-to-bed ratio	1	1
Facilities for TPN	5	5
Facilities for pediatric ventilation	8	9
Facilities for oxygen support	1	1
Nurses trained for neonatal surgical intensive care	1	1
Interdisciplinary team management	1	1
Postoperative management by pediatric surgeon and neonatologist	2	2
Operation performed by (licensed specialis) pediatric surgeon	10	11
Surgeon grade/position	2	2
Trained pediatric anesthesiologist	1	1
Surgical safety checklist	1	1
Use of a feeding guideline	1	1
Compliance to guideline usage	1	1
Intestinal rehabilitation program (IRP)	2	2
**Diagnostics**		
Prenatal diagnosis	27	29
Prenatal diagnosis of polyhydramnios	13	14
Antenatal screening	3	3
Age at diagnosis	5	5
Prenatal ultrasound	14	15
Prenatal MRI (fetal MRI)	2	2
MR imaging protocol	1	1
(Offering) Prenatal chromosomal analysis by amniocentesis	2	2
abdominal X-Ray	19	20
Double bubble sign	5	5
Tripple bubble sign (on X-ray)	5	5
Multiple bubble sign	2	2
Multiple air-fluid levels (on X-ray)	3	3
Free air	1	1
Unused colon (on X-ray)	1	1
Dilated bowel loops (on X-ray)	5	5
Gastrographic contrast studies	3	3
Upper gastrointestinal contrast study	2	2
Lower gastrointestinal contrast study	1	1
Contrast enema	2	2

Upper gastrointestinal endoscopy	1	1
Abdominal ultrasound	8	9
Echocardiography	2	2
Chloride sweat test	1	1
Genetic screening for cystic fibrosis	1	1
Genetic counselling	1	1
**Perioperative management**		
Nutritional status preoperatively	3	3
Laboratory investigations	8	9
Monitoring and maintaining correct body temperature	3	3
TPN (total parenteral nutrition)	9	10
nasogastric tube placement	10	11
Nil per os	1	1
intravenous fluid replacement	10	11
Correction of dehydration	2	2
Resuscitation	6	6
Correction of acid-base balance	1	1
Correction and maintenance of blood glucose level	1	1
Correction of anemia before surgery	1	1
Preoperative antibiotics	9	10
Antibiotic treatment on arrival	2	2
Ceftriaxone and metronidazole (preoperative)	1	1
Cephalosporins and metronidazole (at least 10 days)	1	1
Broad-spectrum antibiotics	3	3
Additional surgery	1	1
Prior cardiac surgery	1	1
Combined: surgical technique	15	16
Bowel length (measured during surgery, before resection)	1	1
Central line/peripherally inserted central catheter	8	9
Central line (days) (peripherally inserted and/or surgically placed central catheter and/or umbilical venous catheter)	2	2
Umbilical catheter	1	1
Intravenous access (number of attempts during hospitalization)	1	1
**Operative management**		
Episodes of general anaesthesia	1	1
Type of anesthesia used for primary intervention	1	1
General anaesthesia with endotracheal tube or laryngeal airway	1	1
Intervention without anaesthesia and with or without analgesia	1	1
Local anaesthesia only	1	1
Spinal or caudal anaesthesia	1	1
Ketamine anaesthesia	1	1
Anesthesia administration	1	1
Surgical technique	11	12
Type of procedure (laparotomy or laparoscopy)	21	22
Minimally invasive surgery	1	1

Surgical approach	2	2
Conversion to open surgery	2	2
Operative time of CO2 exposure	1	1
<u>Perforation (peroperative finding)</u>	9	10
<u>Resection and anastomosis</u>	32	34
<u>Resection and end-to-end anastomosis</u>	8	9
Wide proximal resection and anastomosis	2	2
Atretic segment resection	1	1
Single-segment resection with single anastomoses in multiple atresias if total usable small bowel length > 75 cm	1	1
Bowel resection and ileotransverse anastomosis	1	1
<u>Primary anastomosis</u>	27	29
<u>Type of incision</u>	8	9
<u>Location of anastomosis</u>	6	6
distance from the anastomosis to the ileocecal region	1	1
Distance from duodenojejunal flexure to Santulli	1	1
Distance from ileocecal valve to Santulli	1	1
Distance between stomal vent and duodenojejunal junction	1	1
<u>Tapering enteroplasty</u>	18	19
Minimal resection and tapering enteroplasty	2	2
Enteroplasty	2	2
Subacute serial transverse enteroplasty	1	1
Primary tapering of dilated small bowel	4	4
Narrowing of the proximal dilated bowel	1	1
Bowel plication	2	2
Location of bowel plication	1	1
Bowel diameter after plication	1	1
Plication	1	1
Mesoplasty	1	1
<u>Technique anastomosis (e.g. hand sewn, stapler)</u>	9	10
5 mm laparoscopic stapler	1	1
Minimal staple overlap	1	1
Hand sewn (anastomosis)	4	4
Direct anastomosis of the dilated segment to the non-dilated distal segment	1	1
<u>Type of anastomosis (end/end, end/side, side/side, end/side)</u>	15	16
Drainage, enterostomy and radical surgery	1	1
<u>Discrepancy proximal and distal diameter</u>	11	12
Discrepancy in diameter between proximal and distal bowel at anastomosis (ratio)	2	2
diameter ratio of proximal to distal bowels	2	2
Resection of part of antimesenteric wall	1	1
<u>Greater than 4:1 disparity in luminal diameter between proximal and distal intestine</u>	5	5
Luminar size discrepancy present	4	4
Ratio of diameter two bowel ends less than or equal to 4:1	1	1
<u>Type of resection</u>	19	20
<u>Resection of dilated proximal segment</u>	8	9
<u>Small bowel resection</u>	9	10

Small bowel resection with stoma creation	1	1
Small bowel resection without creation of stoma	1	1
Intestinal resection	8	9
Jejunal resection	2	2
Colonic resection	3	3
Perpendicular resection edge at the proximal bowel	1	1
Jejunal plus ileal resection excluding ileocoecal valve	2	2
Jejunal plus ileal resection including ileocoecal valve	2	2
Ileal plus colonic resection including ileocoecal valve	2	2
Bishop-Koop procedure	8	9
Appendectomy	2	2
Memranectomy with anastomosis	1	1
Number of anastomoses	5	5
Length of resected intestine (cm)	6	6
Length of dilated bowel loops	1	1
Length of distal viable intestinal segment	1	1
Time between presentation and surgery	5	5
Time between admission and surgery	2	2
Duration of procedure	24	26
Anesthesia time / duration of anesthesia	1	1
Duration of surgery > 1 hour	1	1
Duration of stoma closure	1	1
Procedure priority	6	6
Timing of surgery (daytime or night time)	3	3
Estimated blood loss	5	5
Suturing technique	9	10
Type of sutures	12	13
Use of absorbable sutures	3	3
Monofilament absorbable sutures (PDS)	1	1
Braided absorbable sutures (Vicryl)	3	3
4-0 vicryl	1	1
Vicryl 5-0 sutures	5	5
6-0 monofilament suture	1	1
Vicryl or polydioxanone sutures	1	1
Temporary/primary stoma	31	33
Type of stoma	4	4
Enterostomy	19	20
Mikulicz double barrel ileostomy	4	4
Double barrel ileostomy	1	1
Temporary ostomy and delayed closure	1	1
Level of intestinal diversion	1	1
Divided stoma	2	2
Bishop-Koop stoma	2	2
Santulli procedure	1	1
Delayed primary anastomosis	1	1
Time between first surgery and secondary ostomy	1	1

**Postoperative**		
Direct bilirubin	6	6
Direct bilirubin >2 mg/dL	4	4
Direct bilirubin > 4 mg/dL	1	1
Highest level (peak) of total bilirubin	2	2
Total bilirubin level at discharge	1	1
Percent time of hospital stay with elevated total bilirubin	1	1
Bilirubin levels at time of definitive surgery	2	2
Albumin	5	5
Platelets (Thrombocyts)	2	2
INR	1	1
Liver function	2	2
Blood grouping	2	2
Complete blood count/full blood count	3	3
Coagulation tests	3	3
Serum electrolytes	6	6
Serum phosphate	1	1
Serum potassium (preoperative)	3	3
Seum sodium	2	2
Serum magnesium	2	2
Seum immunoglobulin levels (IgG, IgM, IgA)	1	1
Hemoglobin of neonate	3	3
Hematocrit of neonate	1	1
Blood investigation	2	2
Leukocyte count	2	2
Kidney function	3	3
CRP preoperatively	2	2
CRP performed within 72 hours of surgery	1	1
CRP performed after 72 hours of surgery	1	1
CSF (cerebrospinal fluid) culture positive	1	1
Alanine aminotransferase (ALAT)	1	1
Aspartate aminotransferase (ASAT)	1	1
Post-surgery catabolic state	1	1
Time to stomal function	2	2
Time to bowel continuity	10	11
(placement of) T-tube enterostomy	2	2
Placement of transanastomotic feeding tube	2	2
Duration of T-tube enterostomy	2	2
Free distal flow of T-tube enterostomy confirmed by contrast study before removal	1	1
Time to closure of T-tube insertion place	2	2
Mucous fistula	1	1
No of antibiotic courses	1	1
Duration of antibiotics	2	2
Need for cardiac specific therapy (diuretic or inotropic drugs)	2	2
Inotrope support	3	3
Intranasal oxygen support	1	1

time of first defecation	2	2
Weight gain during hospital stay	2	2
Treatment with steroids, risk factor for anastomotic complications	3	3
preoperative steroid administration within 30 days of surgery	1	1
Postoperative weight	1	1
Timing of start of parenteral nutrition (age of start, pre- or postoperatively)	6	6
Duration of parenteral nutrition (in days)	19	20
Nutritional status postoperatively	1	1
Dependence on nutritional support	1	1
Type of nutrition administered	1	1
Weight at time of initial feed	1	1
Time to initial postoperative feeding	3	3
Weaning off of PN	3	3
Time to start enteral feeding	15	16
Minimal enteral feeding	1	1
Nasogastric feeding tube	7	7
Nasogastric tube feeding (days)	1	1
Time to full enteral nutrition	23	25
Achievement of full enteral nutrition prior to discharge	2	2
Time to initial oral feeding	15	16
Time to establishment of full oral intake	10	11
Time to reach 50% enteral nutrition	1	1
Time to initiation of feeds after reanastomosis	1	1
Malnutrition	8	9
Feeding intolerance (postoperative)	5	5
Abdominal distension (postoperative)	2	2
Vomiting (postoperative)	3	3
Lactation consultation and support	1	1
Breast milk use	7	7
MOM (Mother's Own Milk) 48 hours prior to hospital dicharge	1	1
Oral sphere stimulation	1	1
Donor breast milk use	2	2
Diet of > 50% breast milk	1	1
Use of age-appropriate formula	1	1
Commercial formula	2	2
Formula feeding at discharge	1	1
Bovine-based HM fortifiers	1	1
1/5th glucose saline (postoperative maintenance fluid)	1	1
Supplementary electrolytes (postoperatively)	1	1
Plasma transfusion on alternate days (postoperatively)	1	1
2-3 g/kg/d of lipid when receiving PN	1	1
Enteral fish oil	1	1
Treatment with phenobarbital	1	1
Treatment with ursodeoxycholine	1	1
Probiotics (for prevention of neonatal gastrointestinal perforation)	1	1
Synbiotics (prevention of perforation)	1	1

Oral antifungal drugs (prevention of perforation)	1	1
Dextrose containing intravenous fluids	1	1
Duration of mechanical ventilation	7	7
Non-home discharge	1	1
Discharge to home	2	2
Discharge to other hospital	2	2
Discharge with a feeding tube	1	1
Duration of follow up	22	23
30-day follow up	1	1
1-year follow-up	8	9
2-year follow-up	3	3
Follow-up contrast studies/radiographs of anastomosis and distal small bowel	2	2

[1] Underlined: mentioned in more than 5% of articles.

[2] **Bold headings:** categories created to organize the variables.
