# Supplementary material for: Building the Foundation for Standardized Care Metrics in Jejunoileal Atresia: A Systematic Review of Reported Baseline Characteristics, Treatment Variables and Outcomes
Source: J Clin Med. 2025 Aug 12;14(16):5693. doi: 10.3390/jcm14165693 (PMC12386392; doi:10.3390/jcm14165693)
Supplement: Supplementary file 1 [file jcm-14-05693-s001.zip › jcm-3752219 - Supplementary table 2 - All identified patient characteristics.pdf]

Table S2. All identified patient characteristics.

**Patient characteristics**	**n**	**%**
Sex	81	86
Gestational age	67	71
Prematurity	33	35
Small for gestational age	5	5
Age at admission (days)	10	11
Age at admission to NICU	1	1
Age at presentation / Delay in presentation	18	19
Age at presentation to pediatrician	1	1
Age at presentation to surgeon	3	3
Age at onset	2	2
Age at referral (days)	1	1
Age at surgery	41	44
Birth weight (grams)	60	64
Birth weight (Z-score)	3	3
WSD score at birth (weight standard deviation)	2	2
Low birth weight (1.5-2.49)	4	4
Extremely low birth weight (ELBW) (<1kg)	2	2
Very low birth weight (1-1.49kg)	1	1
Normal birth weight (2.5-4.2kg)	1	1
Weight at presentation	13	14
Weight at surgery	14	15
Intrauterine growth restriction (IUGR)	4	4
Birth length (cm)	2	2
Birth length (z-scores)	1	1
Birth head circumference (cm)	3	3
Birth head circumference z scores	1	1
Fetal growth (evaluated every 2 weeks starting from diagnosis IA)	1	1
Race (Caucasian, AA, Asian, other)	7	7
Apgar 1 min	7	7
Apgar 5 min	9	10
Apgar 10 min	1	1
Apgar score (total)	3	3
**Paternal characteristics**		
Maternal age	10	11
Maternal alcohol use	1	1
Maternal recreational drugs (cocaine, marijuana)	1	1
Maternal tobacco use	2	2
Maternal acute or chronic diseases during pregnancy	1	1
Pregnancy history	1	1
Primipara vs multipara	5	5
Twin gestation	1	1
Multifetation	1	1
Maternal eclampsia	1	1
placental abruption	1	1
Parental consanguinity	2	2
Type of delivery (vaginal or cesarean)	13	14
Location of delivery	6	6
Asphyxia	2	2
(Mother living in population with) Unmet Basic Needs	1	1
Maternal education	1	1
Maternal sepsis	1	1

Income	1	1
Patient origin	1	1
Insurance status	1	1
Socioeconomic status	2	2
**Preoperative status**		
Clinical condition on arrival (sepsis, hypovolaemia, hypothermia)	3	3
Preoperative perforation	4	4
Ventilator use (at time of operation)	2	2
Oxygen dependence (at time of operation)	2	2
Tracheostomy dependence	1	1
ASA class	7	7
Hemodynamic instability	4	4
Hemodynamic state	1	1
Pneumoperitoneum	1	1
preoperative dependence on nutritional support	1	1
Hypothermia (on arrival)	1	1
Fever (on arrival)	1	1
Hypotension (on arrival)	1	1
Tachycardia (on arrival)	1	1
**Disease characteristics**		
Type of atresia (I: mucosal web, II atretic fibrous cord, IIIa: V-shaped mesenteric defect, type IIIb apple peel atresia, type IV)	35	37
Level of obstruction (duodenal, jejuno-ileal, colonic)	31	33
Site of intestinal atresia	21	22
multiple atresias	14	15
Isolated atresia/stenosis	2	2
atresia/stenosis located at the oral side from Treitz	1	1
atresia/stenosis located at the anal side from Treitz	1	1
Length between atresia and the ligament of Treitz	1	1
**Symptoms**		
Constipation	3	3
Absolute constipation	2	2
Abdominal distension	13	14
Abdominal tenderness	1	1
Failure to pass meconium	8	9
Delayed passage of meconium	1	1
Haematochezia	1	1
Bilious vomiting	9	10
Jaundice	3	3
Vomiting	11	12
Frothing from mouth	1	1
Bleeding per rectum	1	1
Groin swelling	1	1
Loss of thrive	1	1
Failure to thrive	1	1
**Comorbidities**		
Associated anomalies	62	66
Syndrome	9	10
Trisomy 21	13	14
Trisomy 18	1	1
46, XX, del(13)(q22q31)	1	1
Chromosomal abnormalities	8	9
Multiple structural anomalies	1	1
Cardiac anomalies (all anomalies beneath combined*)	38	40
Cardiac risk factors	2	2
Cardiopulmonary abnormalities	4	4

Ventricular septal defect	7	7
Atrial septal defect	3	3
Atrioventricular septal defect	1	1
Patent ductus arteriosus	6	6
Patent foramen ovale	1	1
Aortic valve stenosis	2	2
Pulmonary stenosis	1	1
Common atrium	1	1
Double-outlet right ventricle	1	1
Tetralogy of Fallot	3	3
Transposition of the great arteries	1	1
Total anomalous pulmonary venous connection	1	1
Tricuspid atresia	1	1
Hypoplastic aortic arch	1	1
Hypoplastic left heart syndrome	1	1
Dextrocardia	1	1
Status post repair of congenital heart defect	1	1
Residual hemodynamic abnormality with or without medications	1	1
Cyanotic heart disease	1	1
Ventricular dysfunction requiring medication	1	1
Respiratory anomalies (combination of all respiratory anomalies mentioned*)	16	17
Respiratory anomalies	2	2
Chronic lung disease	1	1
Tracheomalacia	2	2
Laryngomalacia	1	1
Tracheostenosis	2	2
Subglottic stenosis	1	1
Upper airway obstruction or stenosis	1	1
Hypoplastic lung	1	1
Congenital pulmonary airway malformation	1	1
Apnea of prematurity	1	1
Wet lung	1	1
Persistent pulmonary hypertension	2	2
Bronchopulmonary dysplasia	3	3
Congenital diaphragmatic hernia	3	3
Cystic fibrosis	8	9
Gastrointestinal anomalies (combination of all gastrointestinal anomalies mentioned*)	28	9
Gastrointestinal anomalies	8	3
Tracheoesophageal fistula	6	6
Esophageal atresia	6	6
Congenital short bowel syndrome	3	4
Duodenal atresia	5	5
Malrotation of intestine	13	14
Gastric duplication	1	1
Jejunal duplication	1	1
Meckel diverticulum	6	6
Pyloric stenosis	1	1
Biliary atresia	3	3
Galbladder agenesis	1	1
Gallbladder duplication	1	1
Gastroesophageal reflux	1	1
Volvulus	9	10
Intussusception	2	2
M. Hirschsprung	4	4
Abdominal wall defects (combination of all abdominal wall defect mentioned*)	7	7
Omphalocele	4	4
Gastroschisis	7	7

Single umbilical artery	1	1
Umbilical cord ulcer	1	1
Abdominal wall defect	4	4
Preduodenal portal vein	1	1
Portosystemic shunt	1	1
Situs inversus	1	1
Musculoskeletal anomalies (combination of all musculoskeletal anomalies mentioned*)	14	15
Muculoskeletal	4	4
Vertebral anomalies	4	4
Anomaly of digit	1	1
Absent radius	1	1
Radial anomalies	1	1
Anomaly of ribs	1	1
Hyaline membrane disease	1	1
Supernumerary digits/polydactyly	3	3
Syndactyly	2	2
Periauricular skin tag	1	1
Absent toes	1	1
Orthopedic anomalies	1	1
Dysmorphic features	1	1
Club feet	1	1
Limb defects	2	2
Renal anomalies	9	10
Horseshoe kidney	1	1
Pelvic kidney	1	1
Hydronephrosis	2	2
Hydroureter	1	1
Double ureter	1	1
Unilateral renal agenesis	2	2
Renal cyst	1	1
Urogenital anomalies (combination of all urogenital anomalies mentioned*)	22	23
Disorder of sex development	2	2
Urogenital anomalies	4	4
Cryptorchism	1	1
Hypospadia	4	4
Ambiguous genitalia	2	2
Anal atresia	4	4
Imperforate anus	1	1
Vestibular anus	1	1
Persistent cloaca	1	1
Anorectal malformation	12	13
High anorectal malformation	1	1
Central Nervous System (all anomalies concerning the central nervous system combined*)	15	16
Central nervous system	5	5
Neurological anomalies	1	1
Impaired cognitive status	1	1
Hypoxic ischemis encephalopathy	1	1
Stroke	1	1
Myelomeningocele	1	1
Cerebellar hypoplasia	1	1
Microcephaly	1	1
Agenesis of corpus callosum	1	1
Intraventricular hemorrhage	2	2
Intracerebral hemorrhage related to neonatal immaturity	3	3
Auditory neuropathy	1	1
Retinopathy of prematurity	1	1
Schizencephaly	1	1

Spina bifida	1	1
Lipoma of filum terminale spinal part	1	1
Seizures	1	1
Cerebral palsy	1	1
Neuromuscular disorder	1	1
Dandy Walker malformation	1	1
Foetal alcohol syndrome	1	1
**Other anomalies**		
VACTERL association	5	5
Annular pancreas	8	9
Polysplenia	1	1
Asplenia	1	1
Deafness	1	1
Inner ear hypoplasia	1	1
Ocular anomaly	1	1
Cleft lip and palate	4	4
Head and neck	1	1
Craniomaxillofacial deformity	1	1
Craniosynostosis	1	1
Odd appearance	1	1
Malformation of the auricle	1	1
Accessory auricle	1	1
Facial cleft	1	1
Fistula auris congenita	1	1
Obstructio ductus nasolacrimalis	1	1
Hemangioma	1	1
Unclassified vascular anomaly	1	1
Thrombocytopenia	1	1
Anemia of prematurity	1	1

Underlined: mentioned in more than 5% of articles.

Bold headings: categories created to organise the variables.
