# Supplementary material for: Building the Foundation for Standardized Care Metrics in Jejunoileal Atresia: A Systematic Review of Reported Baseline Characteristics, Treatment Variables and Outcomes
Source: J Clin Med. 2025 Aug 12;14(16):5693. doi: 10.3390/jcm14165693 (PMC12386392; doi:10.3390/jcm14165693)
Supplement: Supplementary file 1 [file jcm-14-05693-s001.zip › jcm-3752219 - Supplementary table 1 - individualized overview of included studies and study characteristics.pdf]

Table S1. Individualized overview of included studies and study characteristics.

Author	Title	Year	Continent	*n*	Study design		
**Elsinga, R. M., et al**	Motor and cognitive outcome at school age of children with surgically treated intestinal obstructions in the neonatal period	2013	Europe	10	Observational	Prospective	Cohort
**Nusinovich, Y., et al**	Long-term outcomes for infants with intestinal atresia studied at Children's National Medical Center	2013	North America	63	Observational	Retrospective	Cohort
**Correa, C., et al**	Congenital malformations of pediatric surgical interest: prevalence, risk factors, and prenatal diagnosis between 2005 and 2012 in the capital city of a developing country. Bogota, Colombia	2014	South America	14	Observational	Retrospective	Cohort
**Ezomike, U. O., et al**	Outcomes of surgical management of intestinal atresias	2014	Africa	13	Observational	Retrospective	Cohort
**Muhammad ZafaR Iqbal, et al**	Neonatal intestinal obstruction an analysis of hospital data at pediatric surgery department Sheikh Zayed Hospital, Rahim Yar Khan	2014	Asia	11	Observational	Prospective	Cohort
**Takahashi D, et al**	Population-based study of esophageal and small intestinal atresia/stenosis	2014	Asia	56	Observational	Retrospective	Cohort
**Yuki Tsuji, et al**	A new paradigm of scarless abdominal surgery in children: transumbilical minimal incision surgery	2014	Asia	5	Comparative	Retrospective	Cohort
**Jun Wang, et al**	Prolonger feeding difficulties after surgical correction of intestinal atresia: a 13-year experience	2014	Asia	81	Observational	Retrospective	Cohort
**Bidondo M.P, et al**	Study on the prevalence and neonatal lethality in patients with selected congenital anomalies as per the data of the National Registry of Congenital Anomalies of Argentina	2015	South America	218	Observational	Retrospective	Cohort
**Mohinuddin, S., et al**	Outcomes of full-term infants with bilious vomiting: Observational study of a retrieved cohort	2015	Europe	12	Observational	Retrospective	Cohort
**Yagi, M., et al**	Twenty-year trends in neonatal surgery based on a nationwide Japanese surveillance program	2015	Asia	1475	Observational	Retrospective	Cohort
**Al-Zaiem M, et al**	Use of T-tube enterostomy in neonatal gastro-intestinal surgery	2016	Asia	34	Observational	Retrospective	Cohort
**Christine Burgmeier, et al**	The Role of Laparoscopy in the Acute Neonatal Abdomen	2016	Europe	8	Observational	Retrospective	Cohort
**F. Fredriksson, et al**	Adhesive small bowel obstruction after laparotomy during infancy	2016	Europe	40	Observational	Cross-sectional	Cohort
**Shilpi Gupta, et al**	Intestinal Atresia: Experience at a Busy Center of North-West India	2016	Asia	78	Observational	Retrospective	Cohort

**Kamal Nain Rattan, et al**	Modified Gut Anastomotic Technique in Type III and Type IV Jejunoileal Atresias	2016	Asia	15	Observational	Retrospective	Cohort
**Kate B. Savoie, et al**	Standardization of Feeding Advancement After Neonatal Gastrointestinal Surgery: Does It Improve Outcomes?	2016	North America	25	Comparative	Retrospective	Cohort
**S Shetty, et al**	Length of stay and cost analysis of neonates undergoing surgery at a tertiary neonatal unit in England	2016	Europe	42	Observational	Prospective	Cohort
**Soichi Shibuya, et al**	Factors Conducive to Catch-Up Growth in Postoperative Jejunoileal Atresia Patients as Prognostic Markers of Outcome	2016	Asia	42	Comparative	Retrospective	Cohort
**Julia K. Shinnick, et al**	Effects of a Breast Milk Diet on Enteral Feeding Outcomes of Neonates with Gastrointestinal Disorders	2016	North America	54	Comparative	Retrospective	Cohort
**Vijay Singh, et al**	Congenital Neonatal Intestinal Obstruction: Retrospective Analysis at Tertiary Care Hospital	2016	Asia	27	Observational	Retrospective	Cohort
**Anjali Verma, et al**	Neonatal Intestinal Obstruction: A 15 Year Experience in a Tertiary Care Hospital	2016	Asia	148	Observational	Retrospective	Cohort
**Fanny Yeung, et al**	Early Reoperations after Primary Repair of Jejunoileal Atresia in Newborns	2016	Asia	43	Observational	Retrospective	Cohort
**Yoon Jung Boo, et al**	Comparison of surgical outcomes among infants in neonatal intensive care units treated by pediatric surgeons versus general surgeons: The need for pediatric surgery specialists	2017	Asia	31	Comparative	Retrospective	Cohort
**Regla C. Broche-Candó, et al**	Neonatal Surgery Case Fatality and Associated Factors in a Cuban Pediatric Hospital	2017	Central America	37	Observational	Retrospective	Cohort
**Sarah Cairo, et al**	Disparity in access and outcomes for emergency neonatal surgery:intestinal atresia in Kampala, Uganda	2017	Africa	60	Comparative	Retrospective	Cohort
**Kin Wai Edwin Chan, et al**	Cystic meconium peritonitis with jejunoileal atresia: Is it associated with unfavorable outcome?	2017	Asia	53	Comparative	Retrospective	Cohort
**Sebastian O Ekenze, et al**	Neonatal surgery in a developing country: Outcome of co-ordinated interdisciplinary collaboration	2017	Africa	14	Comparative	Retrospective	Cohort
**T. Erickson, et al**	Impact of hospital transfer on surgical outcomes of intestinal atresia	2017	North America	1672	Comparative	Retrospective	Cohort
**Jessica Gonzalez-Hernandez, et al**	Predicting time to full enteral nutrition in children after significant bowel resection	2017	North America	14	Observational	Retrospective	Cohort
**Mustefa Mohammed, et al**	Intestinal Obstruction in Early Neonatal Period: A 3-Year Review Of Admitted Cases from a Tertiary Hospital in Ethiopia	2017	Africa	6	Observational	Retrospective	Cohort
**Eva I. Rubio, et al**	Prenatal magnetic resonance and ultrasonographic findings in small-bowel obstruction: imaging clues and postnatal outcomes	2017	North America	9	Observational	Retrospective	Cohort

**Masahito Sato, et al**	Neonatal gastrointestinal perforation in Japan: a nationwide survey	2017	Asia	33	Observational	Retrospective	Cohort
**Fares Al-Jahdali, et al**	Risk Factors and Short Outcome of Bowel Atresia in Neonates at Tertiary Hospital	2018	Asia	11	Comparative	Retrospective	Case-control
**Hizuru Amano a,b, et al**	The impact of body weight on stapled anastomosis in pediatric patients	2018	Asia	43	Comparative	Retrospective	Cohort
**S.B. Cairo, et al**	Mortality after emergency abdominal operations in premature infants	2018	North America	287	Observational	Retrospective	Cohort
**G.C. Hintz, et al**	Stapled versus hand-sewm pediatric anastomoses: a retrospective cohort study	2018	North America	15	Comparative	Retrospective	Cohort
**Annika Mutanen, et al**	Complicated gastroschisis is associated with greater intestinal morbidity than gastroschisis or intestinal atresia alone	2018	Europe	25	Comparative	Retrospective	Cohort
**E. Nakamura, et al**	Retrospective study of umbilical cord ulceration related to congenital intestinal atresia: a single-center report	2018	Asia	20	Comparative	Retrospective	Cohort
**K. Otake, et al**	Clinical factors associated with in-hospital death in pediatric surgical patients admitted to the neonatal intensive care unit: a 15-year single tertiary center experience	2018	Asia	56	Observational	Retrospective	Cohort
**Y. Peng, et al**	Is the Bishop-Koop procedure useful in severe jejunoileal atresia?	2018	Asia	41	Observational	Retrospecive	Cohort
**T.T. Sholadoye, et al**	Presentation and outcome of treatment of jejunoileal atresia in Nigeria	2018	Africa	38	Observational	Retrospective	Cohort
**Patrick C Bonasso, et al**	24-hour and 30-day perioperative mortality in pediatric surgery	2019	North America	2559	Observational	Retrospective	Cohort
**C. Dingemann, et al**	Impact of maternal eduacation on the outcome of newborns requiring surgery for congenital malformations	2019	Europe	24	Comparative	Retrospective	Cohort
**Margot M. Hillyer, et al**	Primary versus secondary anastomosis in intestinal atresia	2018	North America	92	Comparative	Retrospective	Cohort
**C. Jarkman, et al**	Predictive factors for postoperative outcome in children with jejunoileal atresia	2019	Europe	47	Observational	Retrospective	Cohort
**A.E. Joda, et al**	Outcomes of end-to-side oblique anastomosis as a surgical technique for jejuno-ileal atresia	2019	Asia	34	Observational	Prospective	Cohort
**J.D. Kauffman, et al**	Risk factors for adverse outcomes after ostomy reversal in infants less than six months old	2019	North America	241	Observational	Retrospective	Cohort
**Illya Martynov, et al**	The outcome of Bishop-Koop procedure compared to divided stoma in neonates with meconium ileus, congenital intestinal atresia and necrotizing enterocolitis	2019	Europe	31	Observational	Retrospective	Case-control
**Noriyuki Nakamura. et al**	Umbilical Cord Ulcer and Intrauterine Death in Fetal Intestinal Atresia	2019	Asia	19	Observational	Retrospective	Cohort

**O.O. Ogundoyin, et al**	Outcome of management of neonatal intestinal obstruction at a tertiary center in Nigeria	2019	Africa	6	Observational	Retrospective	Cohort
**Y. Peng, et al**	Comparison of outcomes following three surgical techniques for patients with severe jejunoileal atresia	2019	Asia	105	Comparative	Retrospective	Cohort
**N. Sharma, et al**	Transanastomotic tube in intestinal atresia: how beneficial are they?	2019	Asia	46	Comparative	Retrospective	Cohort
**Casey T. Walk, et al**	Neonatal Intestinal Anastomosis Using a 5mm Laparoscopic Stapler	2019	North America	6	Observational	Retrospective	Cohort
**S. Yang, et al**	Bowel plication in neonatal high jejunal atresia	2019	Asia	43	Comparative	Retrospective	Cohort
**H. Zhu, et al**	Long-term surgical outcomes of apple-peel atresia	2019	Asia	39	Observational	Retrospective	Cohort
**N. Almajali, et al**	Neonatal intestinal obstruction in Jordan: A single center 6 year experience	2019	Asia	9	Observational	Retrospective	Cohort
**Vamsi Batta, et al**	Early neurodevelopmental outcomes of congenital gastrointestinal surgical conditions: a single-centre retrospective study	2020	Oceania	16	Observational	Retrospective	Cohort
**L.C. Dewberry, et al**	Is tapering enteroplasty an alternative to resection of dilated bowel in small intestinal atresia?	2020	North America	47	Comparative	Retrospective	Cohort
**R. Hoban, et al**	Supplementation of Mother's own milk with donor milk in infants with gastroschisis or intestinal atresia: a retrospective study	2020	North America	61	Observational	Retrospective	Cohort
**Hansraj Mangray, et al**	Jejuno-ileal atresia: its characteristics and peculiarities concerning apple peel atresia, focused on its treatment and outcomes as experienced in one of the leading South African academic centres	2019	Africa	34	Observational	Retrospective	Cohort
**R.T. Saggers, et al**	An analysis of neonates with surgica diagnoses admitted to the neonatal intensive care unit at Charlotte Maxeke Johannesburg Academic Hospital, South Africa	2020	Africa	46	Observational	Retrospective	Cohort
**B. Thapa, et al**	Patterns and outcome of neonatal intestinal obstruction in Kanti Children's hospital	2020	Asia	30	Observational	Retrospective	Cohort
**S.J. Ullrich, et al**	Burden and outcomes of neonatal surgery in Uganda: results of a five-year prospective study	2019	Africa	93	Observational	Retrospective	Cohort
**Global PaedSurg Research Collaboration**	Mortality from gastrointestinal congenital anomalies at 264 hospitals in 74 low-income, middle-income and high-income countries: a multicentre, internation, prospective cohort study	2021	Europe	681	Observational	Prospective	Cohort
**Ade Gangadhar**	Neonatal small bowel obstruction: pattern, symptoms and diagnostic evaluation: a prospective study	2021	Asia	110	Observational	Prospective	Cohort
**Mario Lima, Neil Di Salvo, et al**	Laparoscopy-assisted versus open surgery in treating intestinal atresia: single center experience	2021	Europe	47	Comparative	Retrospective	Cohort

**Andrea Schemdding, et al**	Jejunoileal atresia: a national cohort study	2021	Europe	435	Observational	Retrospective	Cohort
**Prasanta Kumar Tripathy and Pradeep Kumar Jena**	Demographic Pattern, Management, and Outcome of Intestinal Atresias Among Neonates in a Tertiary Care Indian Hospital	2021	Asia	116	Observational	Retrospective	Cohort
**N. Vinit, D. Mitanchez, et al**	How can we improve perinatal care in isolated multiple intestinal atresia? A retrospective study with a 30-year literature review	2021	Europe	7	Observational	Retrospective	Cohort
**Abeer Aboalazayem, et al**	Outcome of Tapering Enteroplasty in Managing Jejunoileal Atresia	2022	Asia	41	Comparative	Retrospective	Cohort
**Jordan C. Apfeld, et al**	Benchmarking utilization, length of stay, and complications following minimally invasive repair of major congenital anomalies	2022	North America	49	Observational	Retrospective	Cohort
**Yang Chen, et al**	The multivariate cox regression model for complete enteral nutrition after primary anastomosis in neonates with intestinal atresia	2022	Asia	163	Observational	Retrospective	Cohort
**James R. Davis, et al**	Predictors of Survival: A Retrospective Review of Gastroschisis and Intestinal Atresia in Rwanda	2022	Africa	20	Observational	Retrospective	Cohort
**Laurens D. Eeftinck Schattenkerk, et al**	Treatment of jejunoileal atresia by primary anastomosis or enterostomy: Double the operations, double the risk of complications	2021	Europe	80	Comparative	Retrospective	Cohort
**Lindsay A. Gil, et al**	Perioperative outcomes in minimally-invasive versus open surgery in infants undergoing repair of congenital anomalies	2022	North America	95	Comparative	Retrospective	Cohort
**Jinbao Han, et al**	The role of preserved bowel and mesentery fixation in apple-peel intestinal atresia	2022	Asia	42	Observational	Retrospective	Cohort
**Takahiro Hosokawa, et al**	Incidence of late severe intestinal complications after bowel atresia/ stenosis	2022	Asia	27	Observational	Retrospective	Cohort
**Asad Iqbal, et al**	The Success of the Bishop-Koop Surgery in Newborns with Meconium Ileus and Congenital Intestinal Atresia When Compared to Divided Stomas	2022	Asia	35	Comparative	Retrospective	Cohort
**Ishrat Mahtam, et al**	The Outcome of Different Surgical Conditions in Neonates at A Tertiary Care Hospital: A Cross-Sectional Study	2022	Europe	7	Observational	Retrospective	Cohort
**Yoichi Nakagawa, et al**	Circumumbilical incision for neonatal abdominal surgery: additional skin incision when there is difficulty in manipulating the intestine	2022	Asia	58	comparative	Retrospective	Case-control
**Hayssam Rashwan and Mostafa Kotb**	T-tube enterostomy in the management of apple-peel atresia: A case series from a single center	2022	Africa	12	Observational	Prospective	Cohort

**Nicolas Vinit, et al**	Santulli Procedure Revisited in Congenital Intestinal Malformations and Postnatal Intestinal Injuries: Preliminary Report of Experience	2022	Europe	21	Observational	Retrospective	Cohort
**Jiepin Wang, et al**	Evaluation of postoperative feeding strategies in children with intestinal atresia: A single-center retrospective study	2022	Asia	32	Comparative	Retrospective	Cohort
**Ming Yue, et al**	The safety and effectiveness of Santulli enterostomy in neonatal intestinal conditions	2022	Asia	22	Comparative	Retrospective	Case-control
**Zebing Zheng, et al**	Comparison of Hand-Sewn with Stapled Anastomosis in Neonatal Intestinal Atresia Surgery: A Randomized Controlled Study	2022	Asia	82	Comparative	Prospective	Randomized controlled trial
**H du Preez, et al**	The profile and outcome of small bowel atresia at Universitas Academic Hospital	2023	Africa	59	Observational	Retrospective	Cohort
**Adrian Chi Heng Fung, et al**	Primary anastomosis is the preferred surgical approach for proximal intestinal atresia: a retrospective 20-year analysis	2023	Asia	62	Comparative	Retrospective	Cohort
**Mazen Kurdi, et al**	Antimesenteric sleeve tapering enteroplasty with end-to-end anastomosis versus primary end-to-side anastomosis for the management of jejunal/ileal atresia	2023	Africa	57	Comparative	Retrospective	Cohort
**Yuichiro Miyake, et al**	The impact of intestinal atresia on educational and mental health outcomes in school-aged children: A case–control cohort study	2023	North America	94	Comparative	Retrospective	Case-control
**Stella Nimanya, et al**	Surgical neonates in a low-resource setting: Baseline nutrition and outcome assessment	2023	Africa	13	Observational	Prospective	Cohort
**Henrik Røkkum, et al**	Perioperative and Long-Term Outcome in Patients Treated for Jejunoileal Atresia	2023	Europe	70	Observational	Retrospective	Cohort
**Florent Tshibwid A. Zeng, et al**	Gastrointestinal congenital malformations: a review of 230 cases at Albert Royer National Children's Hospital Center in Senegal	2023	Africa	5	observational	Cross-sectional	Cohort
**Florent Tshibwid A Zeng, et al**	Factors associated with mortality in congenital malformations of the gastrointestinal tract in a tertiary center in Senegal	2023	Africa	5	Observational	Retrospective	Cohort
