# Supplementary material for: Building the Foundation for Standardized Care Metrics in Jejunoileal Atresia: A Systematic Review of Reported Baseline Characteristics, Treatment Variables and Outcomes
Source: J Clin Med. 2025 Aug 12;14(16):5693. doi: 10.3390/jcm14165693 (PMC12386392; doi:10.3390/jcm14165693)
Supplement: Supplementary file 1 [file jcm-14-05693-s001.zip › jcm-3752219 - Supplementary material S1 - Search strategy.pdf]

Supplementary material S1 – Search strategy

Database searched	Platform	Years of coverage	Records	Records after duplicates removed
Medline ALL	Ovid	1946 - Present	296	294
Embase	Embase.com	1971 - Present	508	304
Cochrane Central Register of Controlled Trials	Wiley	1992 - Present	22	20
**Total**			**826**	**618**

No other database limits were used than those specified in the search strategies

"What are possible structure, process, and outcome indicators that can be used to improve the quality of care for patients with intestinal atresia?"

Embase 508

('intestine atresia'/de OR 'jejunum atresia'/de OR 'jejunoileal atresia'/de OR (((intestin* OR jejunum* OR jejunoileal*) NEAR/3 (atresi*))):ab,ti,kw) **AND** ('treatment outcome'/exp OR mortality/exp OR morbidity/exp OR ' short bowel syndrome'/de OR survival/exp OR reoperation/exp OR hospitalization/exp OR ' hospital readmission'/exp OR complication/exp OR 'quality of life'/exp OR (outcome* OR mortalit* OR ((short) NEAR/6 (bowel) NEAR/6 (syndrom*)) OR surviv* OR reoperat* OR re-operat* OR hospitalization* OR hospitalisation* OR readmi* OR re-admi* OR complicat* OR quality-of-life* OR QoL OR HRQoL OR HRQL):ab,ti,kw) NOT ('case report'/de OR 'case study'/de OR (case-report* OR case-serie*):ti) NOT ([Conference Abstract]/lim OR [Conference Review]/lim OR [editorial]/lim) AND [2013-2030]/py

Medline

(Intestinal Atresia/ OR (((intestin* OR jejunum* OR jejunoileal*) ADJ3 (atresi*))).ab,ti,kf.) **AND** (exp Treatment Outcome/ OR exp Outcome Assessment, Health Care/ OR exp Mortality/ OR Mortality.fs. OR exp Morbidity/ OR Short Bowel Syndrome/ OR Survival/ OR Reoperation/ OR Hospitalization/ OR Patient Readmission/ OR complications.fs. OR exp Quality of Life/ OR (outcome* OR mortalit* OR ((short) ADJ6 (bowel) ADJ6 (syndrom*)) OR surviv* OR reoperat* OR re-operat* OR hospitalization* OR hospitalisation* OR readmi* OR re-admi* OR complicat* OR quality-of-life* OR QoL OR HRQoL OR HRQL).ab,ti,kf.) NOT (Case Reports/ OR (case-report).ti.) NOT (news OR congres* OR abstract* OR book* OR chapter* OR dissertation abstract*).pt. NOT (editorial).pt. AND 2013:2030.(sa_year).

Cochrane limited to 2013-2023

((((intestin* OR jejunum* OR jejunoileal*) NEAR/3 (atresi*))):ab,ti) **AND** ((outcome* OR mortalit* OR ((short) NEAR/6 (bowel) NEAR/6 (syndrom*)) OR surviv* OR reoperat* OR re-operat* OR hospitalization* OR hospitalisation* OR readmi* OR re-admi* OR complicat* OR quality-of-life* OR QoL OR HRQoL OR HRQL):ab,ti) NOT "conference abstract":kw
