# Supplementary material for: Building the Foundation for Standardized Care Metrics in Jejunoileal Atresia: A Systematic Review of Reported Baseline Characteristics, Treatment Variables and Outcomes
Source: J Clin Med. 2025 Aug 12;14(16):5693. doi: 10.3390/jcm14165693 (PMC12386392; doi:10.3390/jcm14165693)
Supplement: Supplementary file 1 [file jcm-14-05693-s001.zip › jcm-3752219 - Supplementary Table 4 - All identified outcomes.pdf]

Table S4. All identified outcomes.

**Outcomes**	**n**	**%**
Survival rate / mortality	66	70
Survival without complications	1	1
24-hour (perioperative) mortality	3	3
Survival at NICU discharge	2	2
Survival at hospital discharge	4	4
Anesthesia related mortality	1	1
30-day mortality	6	6
Survival at 28 days after birth	1	1
In-hospital mortality (death before discharge)	13	14
Long-term survival	3	3
1-year mortality	3	3
Palliation	1	1
Stoma related complications	9	10
Stomal prolapse	6	6
Remaining small bowel length	17	18
Length of remaining colon	2	2
Resected bowel length	1	1
Percentage of remaining small bowel	4	4
Percentage of remaining colon	1	1
Age adjusted small bowel length	1	1
Age adjusted colon length	1	1
Bowel length (measured during surgery, before resection)	1	1
Stoma bleeding	2	2
Stomal infection	2	2
High output stoma	5	5
Stenosis stoma	3	3
Stoma stricture	2	2
Parastomal hernia	2	2
Peristomal skin irritation	2	2
Peristomal excoriation	2	2
Peristomal abscess	1	1
Peristomal fistula	1	1
Mucocutaneous separation	1	1
Stoma necrosis	2	2
Stoma leakage (causing peritonitis)	1	1
Stoma perforation	1	1
Stoma retraction	2	2
Twisting of stoma	1	1
Stoma revision	1	1
Age adjusted small bowel length	1	1
Length of hospital stay	52	55
Postoperative length of stay	5	5

Length of NICU stay	2	2
Length of PICU stay	1	1
Ileocecal valve still present?	6	6
Serial Transverse enteroplasty procedure (STEP)	2	2
Intestinal lengthening procedure	1	1
No of antibiotic courses	1	1
Postoperative weight	1	1
Bacterial overgrowth	1	1
Growth insufficiency	1	1
Bowel lengthening surgery (STEP, Bianchi)	3	3
Complications	50	53
Postoperative complications	21	22
Preoperative complications	1	1
Perioperative complications	1	1
Iatrogenic injuries	1	1
Intraoperative complications	2	2
Neurologic complications	1	1
Hematologic complications	1	1
Complication grade	1	1
Early (<28 days) complication rate	1	1
Short term complications (< 30 days)	7	7
Late complications	1	1
Late intestinal complications	1	1
Long term complications (> 30 days)	3	3
In-hospital complications	1	1
Time between surgery and day of complication	1	1
Uneventful recovery	3	3
Bleeding	3	3
Bleeding from the liver	1	1
Blood transfusion within 48 hours post surgery	2	2
Blood transfusion	3	3
bleeding requiring blood transfusion <72 h from surgery	1	1
Anemia requiring transfusion	4	4
Anemia	2	2
Cardiac complications	2	2
Cardiac failure	1	1
Endocarditis	1	1
Peritonitis	4	4
Bowel perforation with significant contamination	3	3
Intestinal perforation	6	6
Postoperative perforation	3	3
Intestinal perforation at a non-anastomotic site	1	1
Ultrasound drainage (for anastomotic leakage)	1	1
Insertion of peritoneal drain	2	2
Uncertainty of bowel vitality	1	1
Persistent abdominal distension	1	1

Intestinal dilatation	1	1
Abdominal abscess	2	2
Abdominal collection	1	1
Ascites	1	1
Pseudocyst	2	2
Duplication cyst	2	2
Intestinal hematoma	1	1
Intraabdominal calcification	3	3
Gangrene	2	2
Intestinal necrosis	7	7
Ischemia (per-operative, primary surgery)	5	5
Bowel health compromised	1	1
Pallor (per-operative, primary surgery)	1	1
Discoloration (per-operative, primary surgery)	1	1
Friability of bowel (per-operative, primary surgery)	1	1
Condition of the bowel (per-operatively)	1	1
Mesenteric angiodysplasia	1	1
Abdominal compartment syndrome	1	1
Episodes of meconium peritonitis	11	12
Meconium plug	1	1
Meconium ileus	6	6
Perforated meconium ileus	1	1
Ileus	7	7
Adhesive ileus	3	3
Duration of ileus	2	2
Anastomotic leakage	36	38
Anastomotic bleeding	1	1
Anastomotic dehiscence	2	2
Anastomotic fistula	1	1
Prolonged anastomosis dysfunction	1	1
Anastomotic insufficiency	1	1
Anastomotic complications	2	2
Dismantling of anastomosis and stoma creation	1	1
Anastomotic stricture	14	15
Anastomotic stenosis	5	5
Anastomotic obstruction	1	1
Bowel obstruction	12	13
Adhesive small-bowel obstruction	16	17
Functional obstruction	3	3
Intestinal occlusion	1	1
Intestinal stenosis	1	1
Intestinal stricture	1	1
Prolonged impaired gastrointestinal motility	2	2
Fever	2	2
Infection	9	10
Surgical site infection	11	12

Wound infection	20	21
Wound dehiscence	12	13
Wound discharge	1	1
Wound complications	4	4
Wound rupture	1	1
Wound revision	1	1
Incisional hernia	5	5
Active infection (risk factor anastomotic leakage)	1	1
Drainage for postoperative wound infection	3	3
Pleural drainage due to dislocation of a percutaneous CVC	1	1
Respiratory infection	17	18
Pneumonia	7	7
Aspiration pneumonitis	6	6
Respiratory viral infection	1	1
Pneumonitis	1	1
Pulmonary infection	2	2
Pulmonary complications	1	1
Pulmonary fibrosis	1	1
Respiratory failure	5	5
Respiratory complications	1	1
Respiratory distress	3	3
Pneumothorax	2	2
Pulmonary hemorrhage	1	1
Ventilatory support (for aspiration pneumonitis)	1	1
Chronic lung disease	1	1
Obstructed airways	1	1
Apnea	1	1
Urinary tract infection	5	5
Skin excoriation around insertion site of T-tube enterostomy	3	3
Central nervous system infections	1	1
Hospitalization frequency	1	1
Systemic complications	1	1
Viral infection	1	1
Bacteremia	1	1
Catheter infection	1	1
Sepsis	44	47
Culture proven sepsis	1	1
Number of culture-proven sepsis episodes	2	2
Late onset sepsis	2	2
Late onset sepsis with Gram-negative organisms (after 72 hours of birth)	1	1
Early onset sepsis (within 72 hours of birth)	1	1
Sepsis at presentation	2	2
Sepsis with disseminated intravascular coagulation	2	2
Sepsis from anastomotic leakage	2	2
Apnea with sepsis	1	1
pneumonia with sepsis	1	1

Aspiration with sepsis	1	1
Late catheter sepsis	1	1
Uncontrollable sepsis	3	3
Uncontrollable sepsis from prolonged total parenteral nutrition	1	1
Severe sepsis	1	1
Sepsis with organ failure	1	1
Wound sepsis	2	2
Sepsis with gram-negative organisms	1	1
Sepsis with gram-positive organisms	1	1
Sepsis with multidrug-resistant organisms	1	1
Sepsis with fungal organisms	1	1
Septic shock	7	7
Hypovolemic shock	1	1
Multiple organ dysfunction	3	3
Meningitis	2	2
Purulent meningitis	1	1
Coma (neurologic surgical complication)	1	1
Seizure (neurologic surgical complication)	1	1
Cerebrovascular event (neurological surgical complication)	1	1
Metabolic acidosis	1	1
Metabolic derangements	2	2
Healthcare-associated infection	1	1
healthcare-associated blood stream infection (HABSI)	1	1
Culture positive blood stream infection	2	2
CLABSI (central line-associated bloodstream bloodstream infection)	10	11
Arteriovenous line infection	1	1
TPN-related complications	1	1
Anesthesia related complications	1	1
Unplanned reoperation	38	40
Re-exploration due to bowel perforation	1	1
Reoperation due to bowel dysmotility	1	1
Reoperation (abdominal) within the same admission	1	1
30-day reoperation	4	4
Late (>28 days) abdominal surgical reinterventions	1	1
Reoperation due to missed distal atresia	1	1
Reoperation due to missed jejunal atresia	1	1
Relaparotomy	3	3
Surgical re-exploration and enterostomy	5	5
Re-anastomosis	6	6
Reoperation and side to side anastomosis	1	1
Reoperation/lysis of adhesions owing to adhesive bowel obstructions	11	12
Enterolysis	2	2
Unplanned reintubation	2	2
Number of reoperations	5	5
Number of operations	6	6
Need for more than one surgery	1	1

Total number of laparotomies	1	1
Autologous intestinal reconstruction surgery (AIR)	1	1
Short bowel syndrome	17	18
Central line complication	3	3
Central line thrombosis	1	1
Central line dysfunction	1	1
Dehydration	1	1
Midgut malrotation with volvulus and anastomotic dilatation	1	1
Volvulus	4	4
NEC	7	7
Post-surgical NEC	9	10
Post-discharge onset of NEC	1	1
Intestinal failure	3	3
Malabsorption	3	3
Chronic diarrhea	1	1
Diarrhea (postoperative complication)	1	1
Development of PNALD (parenteral nutrition associated liver dysfunction)	5	5
Hepatic cholestasis	10	11
TPN induced cholestasis	2	2
Biopsy-supported diagnosis of TPN cholestasis	1	1
Liver failure	9	10
Liver disfunction	1	1
Liver insufficiency	1	1
Intestinal failure associated liver disease (IFALD)	2	2
Acute renal insufficiency	2	2
Hepatorenal syndrome	1	1
Congestive heart failure without sepsis	1	1
Organ transplant (liver, bowel)	2	2
Liver transplantation	1	1
Blood clots	1	1
Vena cava thrombosis	1	1
Deep venous thrombosis	2	2
Pulmonary embolism	1	1
Readmission	7	7
30-day readmission	3	3
Unplanned readmission in 1 year	1	1
Number of readmissions	1	1
Readmission to NICU	1	1
**Follow-up**		
growth in height	6	6
Catch-up growth	1	1
Growth in weight	6	6
BMI (follow-up)	1	1
Growth	7	7
Enteral autonomy	2	2
Nutritional habits	1	1

Frequency of bowel motion	1	1
Failure to thrive	1	1
Poor weight gain	1	1
<u>Development</u>	6	6
standardized tests in Grade 3, 7, 8, 9 using the early development instrument	1	1
High school graduation	1	1
suboptimal neurodevelopmental outcomes	1	1
1-year outcomes Griffiths Mental Development Scales (GMDS-II)	1	1
Disability adjusted life years	1	1
Orality disorder	1	1
Satisfaction rate	1	1
long-term gastrointestinal quality of life (QoL)	1	1
Thriving, no complications at 6 months	1	1
Reflux	1	1
<u>G-tube dependent (gastrostomy or gastrojejunostomy dependent)</u>	5	5
Chronic constipation	1	1
Use of laxatives	2	2
Use of proton pump inhibitor	1	1
Anal stenosis	1	1
Anastomotic dilatation	1	1
Chronic abdominal pain	1	1
Hospitalization for abdominal pain	1	1
Infertility	1	1
favorable cosmetic outcomes	1	1
Embarrassment of the scar	1	1
Mild feeding difficulties	1	1
Blindness	1	1
Deafness	1	1
cerebral palsy	1	1
Locomotor performance	1	1
personal and social performance	1	1
hearing and speech	1	1
eye and hand coordination	1	1
Performance	1	1
Movement ABC (Assessment Battery for Children) in Z-score	1	1
Balance	1	1
Fine skills	1	1
Ball skills	1	1
Type of education (formal education, formal education with/without additional educational guidance, special education) at follow up (medial 9,5 years)	1	1
Total IQ	1	1
Verbal IQ	1	1
Performance IQ	1	1
Selective attention	1	1
Attentional control	1	1
Visual perception	1	1

Visuomotor integration	1	1
Verbal memory	1	1
physical health and well-being	1	1
social knowledge and competence	1	1
emotional health/maturity	1	1
language and cognitive development	1	1
general knowledge and communication skills	1	1
Anxiety (ICD)	1	1
Depression (ICD)	1	1
Hyperactivity disorder (ICD)	1	1

[1] Underlined: mentioned in more than 5% of articles.

[2] **Bold headings:** categories created to organize the variables.
